# A Novel Artificial-Intelligence-Based Approach for Classification of Parkinson’s Disease Using Complex and Large Vocal Features

**DOI:** 10.3390/biomimetics8040351

**Published:** 2023-08-07

**Authors:** Rahul Nijhawan, Mukul Kumar, Sahitya Arya, Neha Mendirtta, Sunil Kumar, S. K. Towfek, Doaa Sami Khafaga, Hend K. Alkahtani, Abdelaziz A. Abdelhamid

**Affiliations:** 1 Computer Science and Engineering, Thapar Institute of Engineering and Technology, Patiala 147004, India; 2 Blackstraw Technologies Pvt Ltd., Chennai 160015, India; 3 Graphic Era University, Dehradun 248002, India; 4 Computer Science and Engineering, Chandigarh University, Ajitgarh 140413, India; 5 Department of Computer Science and Artificial Intelligence, SR University, Warangal 506371, India; 6 Department of Computer Science, Graphic Era Hill University, Dehradun 248001, India; 7 Computer Science and Intelligent Systems Research Center, Blacksburg, VA 24060, USA; 8 Department of Communications and Electronics, Delta Higher Institute of Engineering and Technology, Mansoura 35111, Egypt; 9 Computer Sciences, College of Computer and Information Sciences, Princess Nourah bint Abdulrahman University, P.O. Box 84428, Riyadh 11671, Saudi Arabia; dskhafga@pnu.edu.sa; 10 Department of Information Systems, College of Computer and Information Sciences, Princess Nourah bint Abdulrahman University, P.O. Box 84428, Riyadh 11671, Saudi Arabia; 11 Department of Computer Science, College of Computing and Information Technology, Shaqra University, Shaqra 11961, Saudi Arabia; abdelaziz@su.edu.sa; 12 Department of Computer Science, Faculty of Computer and Information Sciences, Ain Shams University, Cairo 11566, Egypt

**Keywords:** neural network, transformer, Parkinson’s disease, dysphonia measures, unbalanced class, tabular data

## Abstract

Parkinson’s disease (PD) affects a large proportion of elderly people. Symptoms include tremors, slow movement, rigid muscles, and trouble speaking. With the aging of the developed world’s population, this number is expected to rise. The early detection of PD and avoiding its severe consequences require a precise and efficient system. Our goal is to create an accurate AI model that can identify PD using human voices. We developed a transformer-based method for detecting PD by retrieving dysphonia measures from a subject’s voice recording. It is uncommon to use a neural network (NN)-based solution for tabular vocal characteristics, but it has several advantages over a tree-based approach, including compatibility with continuous learning and the network’s potential to be linked with an image/voice encoder for a more accurate multi modal solution, shifting SOTA approach from tree-based to a neural network (NN) is crucial for advancing research in multimodal solutions. Our method outperforms the state of the art (SOTA), namely Gradient-Boosted Decision Trees (GBDTs), by at least 1% AUC, and the precision and recall scores are also improved. We additionally offered an XgBoost-based feature-selection method and a fully connected NN layer technique for including continuous dysphonia measures, in addition to the solution network. We also discussed numerous important discoveries relating to our suggested solution and deep learning (DL) and its application to dysphonia measures, such as how a transformer-based network is more resilient to increased depth compared to a simple MLP network. The performance of the proposed approach and conventional machine learning techniques such as MLP, SVM, and Random Forest (RF) have also been compared. A detailed performance comparison matrix has been added to this article, along with the proposed solution’s space and time complexity.

## 1. Introduction

Parkinson’s disease is the second most common age-related neurodegenerative disorder after Alzheimer’s disease. The non-profit group Alzheimer’s Disease International predicts that there will be some 135 million cases worldwide by mid-century, up from 44 million patients today. Similarly, World Health Organization (WHO) predicts that by 2040, as many developed countries’ populations age, neurodegenerative diseases such as Alzheimer’s and other causes of dementia, as well as conditions that affect mainly motor, rather than cognitive, functions, such as Parkinson’s disease (PD) and amyotrophic lateral sclerosis (ALS), will overtake cancer to become the second leading cause of death after cardiovascular disease [1,2,3,4,5].

PD is a progressive nervous system disorder that impairs movement. Symptoms begin gradually, sometimes starting with a barely perceptible tremor in only one hand. Tremors are common, but the disorder also commonly causes stiffness or the slowing of movement [6]. Tremor or shake symptoms manifest themselves in the limb through slowed movement (bradykinesia), making simple tasks difficult and time-consuming. When a patient walks, rigid muscles result in the loss of automatic movements such as blinking, smiling, and swinging of the arms [7]. It affects the patient’s life severely by making social interaction very difficult and worsening their financial condition due to the medical expenses associated with the diagnostics. The average cost of Parkinson’s medication is USD 2500 per year. Parkinson’s-related surgery can cost up to USD 100,000 per patient [8]. As a result, there is an urgent need to develop an effective and affordable method for detecting PD early on in order to avoid the difficulties associated with its severe form and associated expenses.

The difficulty in movement due to rigid muscles affects the patient’s ability to speak and write correctly. Researchers have thoroughly investigated this induced behavior to make a cost-effective and simple screening test to determine the existence of PD. Most datasets are collected in collaboration with hospitals, containing handwritten texts or voice recordings of some predefined sentences. Some collections also include the long phonation of the alphabet. The open-source dataset [9,10] is available in the form of dysphonia measures that contain the extracted features out of the patient’s voice and preserved in the form of a comma-separated values (CSV) sheet. The extracted features also help obscure the patient’s identity and respect the health institution’s confidentiality clause by making subject tracing difficult. Recently, blockchain has emerged as a powerful tool to preserve privacy, and it has also been explored in the healthcare domain [11,12]. Federated learning [13,14] can also be explored for preserving privacy.

In the past, research based on the dysphonia measures was very much limited to a smaller dataset [10,15] with 100 to 300 samples with fewer features; they used classical machine learning approaches such as SVM, RF, and logistic regression. A larger dataset [9] was introduced in 2019 with 756 samples and a substantially larger feature set. We have used this more complex data in our research.

Generally speaking, the SOTA method for complex tabular data uses Gradient Boosting Machines (GBMs), more precisely GBDTs. Due to the immense success of GBDTs and their popularity among ML practitioners, various GBDT-based tools are available, which are well configured to deliver the best accuracy and throughput, such as XgBoost, CaTBoost, LightGBM [16], etc.

Additionally, GBDTs have some limitations that can be resolved by having an NN-based solution, which we have proposed: (a) They are not suitable for continual learning from the data stream. (b) They do not allow end-to-end training of image/text encoders in the presence of multi-modality or tabular data. (c) They are unable to effectively handle noisy and missing data.

In recent years, numerous deep learning solutions have been discovered to address machine learning challenges involving image, text, and audio data. Despite this, very little research has been conducted on the application of deep learning to tabular data. In healthcare, manufacturing, and financial services, tabular data is more prevalent. In the healthcare domain, structurally complex data are more prevalent, and several studies [17,18] have been conducted to address this.

The transformer, a novel NN architecture first introduced by Vaswani et al. [19], has shown promising results in computer vision, natural language processing (NLP), and speech recognition. The usability of the transformer and its variant on the tabular data is not much explored. The current transformer implementation for tabular data such as TabTransformer uses categorical variables only to pass to the transformer blocks. In contrast, continuous variables are projected and merged directly to the output of transformer blocks. In TabTransformer, the authors have provided empirical evidence that transformer-based models are more robust in dealing with missing features than GBDTs.

In this research work, we have first performed a thorough background analysis and literature review. After carefully evaluating their limitations and relevance to our problem statement, we have defined the Vocal Tab Transformer network, which outperforms the current SOTA, i.e., GBDT, in classifying PD and healthy subjects. Specifically, our research proposes a novel transformer-based approach along with a feature selection strategy to identify Parkinson’s disease using the vocal features extracted from the subject’s voice recording. Our method yields at least a 1% higher AUC score than the current SOTA GBDTs models and the precision and recall scores are also improved. We have compared our approach to a more extensive set of ML algorithms such as RF and SVM, along with a detailed analysis of configurations applicable to the proposed solution to determine which factors are more crucial to its performance. Additionally, the Vocal Tab Transformer is compared with MLP to understand the challenges associated with the depth of the model, and it is empirically shown that the transformer-based model performs better with increased depth. Moreover, moving the solution from boosted decision trees to an NN-based solution helps overcome the limitations that come along with it. For example, the shift in the state-of-the-art (SOTA) approach to a neural network (NN) model has opened up new possibilities for creating multi-modal solutions. With the help of this development, tabular datasets can be combined with other image- and voice-based datasets, such as PET/SPECT imaging [20], to produce results with more accuracy and resilience. This innovation opens the door to the development of highly precise and trustworthy multi-modal systems, creating intriguing new research opportunities. This manuscript is structured as follows: To begin, we discuss the work that has been carried out to develop applications that can detect PD using a variety of techniques, as well as the experiments that support this goal. Second, a detailed description of the dataset is provided. Thirdly, we explain and then justify our solution by comparing it to the other SOTA and frequently used methods. Finally, we discuss the implications and consequences of our proposed method and the future scope of work.

## 2. Literature Review

There are many studies in the literature regarding the identification of PD among subjects. In the early days of research related to PD, it is concluded that the voice is the most prominent attribute for diagnosis and the most often affected in the early stages of PD [21]. Improper muscle control can cause the improper production of vowels. This type of speech is easy to use; therefore, it is commonly used in clinical practice for any field of research [22]. Various vocal features have been extracted from the sustained phonation: harmonic-to-noise ratio [23,24,25], jitter, shimmer [23], Mel-spectral frequency coefficients (MFCC) [25,26], the ratio of voiced-to-unvoiced sounds, intelligibility, prosodic features [26], and nonlinear voice features [10]. These features have been used in various ML models for the classification of PD.

Gaffari et al. [27] (2023) have explored a new DL and ML approach to diagnose PD by analyzing speech signals. A novel method called SkipConNet + RF, combining convolutional neural network (CNN) and RF, achieved improved performance, with accuracy rates of 99.11% and 98.30% using voice recordings. Similarly, Nilashi et al. [28] (2023) presented a combined approach using ensemble learning with DBN, Neuro-Fuzzy, EM clustering, PCA, and K-NN to predict the Unified Parkinson’s Disease Rating Scale (UPDRS) in PD diagnosis. The approach improves prediction accuracy and time complexity for large datasets compared to other machine learning techniques. Skaramagkas et al. [29] (2023) have submitted a report that offers a thorough analysis of the deep learning methods applied to PD research between 2016 and January 2023. The paper highlights the potential results of deep learning algorithms in predicting and monitoring PD symptoms based on speech, facial expression, upper limb movement, gait, and these factors combined, but it also draws attention to drawbacks such as data accessibility and model interpretability. According to the study, these issues will be resolved by future developments in deep learning and improved data accessibility, enabling a wider use of this technology in clinical contexts. Anand et al. [30] (2018) have performed a comparative investigation of a wide variety of classification-based ML and DL algorithms with various dimensionality-reduction techniques to differentiate between healthy and diseased individuals. Belic et al. [31] (2019) analyzed 48 relevant studies published in the past, in addition to harmonizing data-gathering techniques, exchanging, and combining data sets. Almeida et al. [32] (2019) evaluated vowel /a/ phonation and the pronunciation of short phrases for PD identification using multiple ML algorithms and discovered that vowel /a/ phonation is more effective. Wroge et al. [33] (2018) investigated the ability of deep NN to reliably diagnose individuals with PD diseases based on their speech recordings, achieving a peak accuracy of 85.5%. Zhang et al. [34] (2018) proposed DeepVoice, a system for detecting PD using mobile-recorded voice. They obtained 90.45 ± 1.71% accuracy with only a 10-second audio clip. Ashour et al. [35] (2020) have worked on the identification of frozen gait for the diagnosis of PD. They have developed an LSTM-based model that significantly outperforms SVM. Balaji et al. [36] (2021) introduced a unique LSTM-based model for detecting the severity rating of Parkinson’s disease using gait patterns. They attained a 98.6% accuracy rate for binary classification and a 96.6% accuracy rate for multiclass classification. Choi et al. compared ML and DL approaches for identifying PD using voice and tap data obtained from cellphones. Wodzinski et al. [37] (2019) calculated the audio spectrum and used a ResNet architecture that was pre-trained for classification and obtained equivalent accuracy to SOTA. Khatamino et al. [38] (2018) have utilized CNN for spirals in handwriting to identify PD. Their recommended method had an 88% success rate. Quan et al. [39] (2021) proposed a bidirectional LSTM model for capturing the time-series dynamic aspects of a speech stream in order to identify PD. This method outperforms conventional machine learning models that employ static features. Xia et al. [40] 2019 proposed a dual-modal deep-learning-based model, where left and right gait is modeled separately by a CNN followed by an attention-enhanced long short-term memory (LSTM) network. Moreover, additional feature processing and selection method on top of a DL model is proven effective in multiple hybrid approaches [41,42,43].

The previously suggested approaches have effectively used DL with data such as voice recordings, handwriting, EEG, and gait patterns, but none of them have examined how to apply deep NN to extracted dysphonia measures from voice recordings. The extracted vocal characteristics also play an important role in concealing the identity of the individual. In this study, we offer a technique for detecting PD utilizing a huge volume of dysphonia measures based on a deep neural network.

## 3. Materials and Methods

### 3.1. Materials

#### 3.1.1. Datasets

In this study, we used the dataset available online at UCI Machine Learning Repository [9] collected at the Department of Neurology in the CerrahpaÅŸa Faculty of Medicine, Istanbul University. We refer to the dataset in this article as the main PD dataset. In comparison to the previously available dataset [10,15], this dataset contains a greater number of samples and is enriched with a variety of new features. It was gathered from 188 patients with PD (107 men and 81 women) with ages ranging from 33 to 87 (65.1 ± 10.9). The control group consisted of 64 healthy individuals (23 men and 41 women) with periods varying from 41 to 82 (61.1 ± 8.9). The data were collected using a microphone set to 44.1 KHz, and following the physician’s examination, the sustained phonation of the vowel /a/ was collected from each subject with three repetitions. All vocal features were derived using various signal-processing algorithms, comprising wavelet transform-based features, baseline features, vocal fold features, TWQT features, and MFCCs features that have been applied to the speech recordings of PD patients to extract important information for PD assessment.

**Figure 1:**
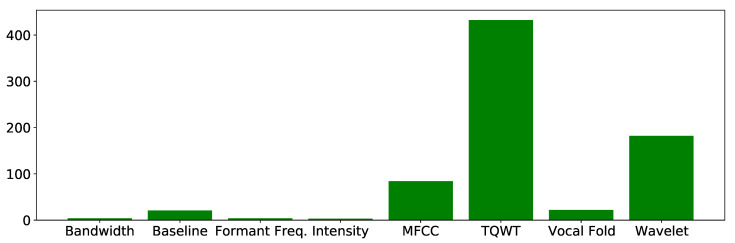
Number of vocal features in each category of dysphonia measure.

**Figure 2:**
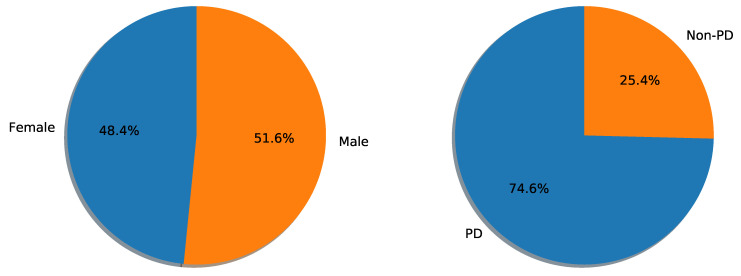
(**Left**): Dataset distribution based on gender. (**Right**): whether the sample belongs to PD or a healthy subject.

The number of features related to each category is shown in Figure 1. It contains a total of 753 unique vocal features along with each patient’s unique ID. This dataset is imbalanced, which means there is a difference in the number of instances of Parkinson’s and non-Parkinson’s patient records. However, the male-to-female ratio is balanced. Refer to Figure 2.

As discussed, other similar PD datasets [10,15] are available, but it has fewer than 300 samples; this is not adequate to train an NN-based model, which requires a substantially larger sample size. Hence, we have used one additional dataset to test the generalizability of the proposed solution:

1. A Parkinson’s speech dataset with multiple types of sound recording [23]. This dataset is not as large as the main PD dataset [9] in terms of subject participation, but it contains a total of 1040 samples; we refer to this dataset in our article as the PD dataset. This dataset [23] contains a total of 40 subjects; half of them had PD, and half of them were healthy. Moreover, the group of PD patients has 6 women and 14 men, while the group of non-PD patients has 10 women and 10 men. Each subject contributed 26 different types of voice recordings, ranging from sustained vowels to short sentences. The number of speech features is quite low compared to the main PD dataset [9], i.e., a total of 26 features.

#### 3.1.2. Data Pre-Processing

As discussed, the main PD dataset contains 756 samples, and each instance has 753 unique features along with the subject’s ID. We have standardized the dataset by applying the following formula to each feature.

(1) $$\begin{array}{c} \chi_{i}^{j}=\frac{x_{i}^{j}-\mu^{j}}{\sigma^{j}} \end{array}$$

where $\mu^{j}$ is the mean and $\sigma^{j}$ is the standard deviation of the selected feature column. $x_{j}^{i}$ and $\chi_{j}^{i}$ are the input and output for the $i^{th}$ row and $j^{th}$ column. Furthermore, the dataset is divided into a ten-fold training and testing set using the stratified k-fold strategy, which helps to obtain the splits with a similar class distribution. Moreover, while splitting the dataset, we made sure that all three samples that belonged to an individual must belong to only one of the sets to maintain the test data’s sanity. A similar data split approach was applied to the PD dataset [23], which contains multiple samples from the same subject, and the subject id was used to carefully define the splits. To address the class-imbalance problem, we used the Adaptive Synthetic (ADASYN) algorithm on the main PD dataset [9]. This is an improved version of SMOTE [44] that oversamples the minority class (non-Parkinson’s) to make it equivalent to the majority class (Parkinson’s). The ADASYN oversampling is applied to the training set of every fold before the models are trained.

### 3.2. Methods

Our solution architecture was inspired by the Transformer first proposed by Vaswani et al. [19] in 2017. A Transformer encoder block consists of a multi-head self-attention layer followed by a position-wise feed-forward layer, with a skip connection (element-wise addition of the input and output of the layer) and normalization being applied after each layer. A self-attention layer contains three parametric matrices known as Key, Query, and Value. Each input embedding is multiplied by the corresponding columns in these matrices to generate their key, query, and value vectors. Formally, let $K\in R^{n*k}$, $Q\in R^{n*k}$, and $V\in R^{n*k}$ be the matrices that contain key, query, and value vectors for each input embedding, where n is the number of features inserted into the network, and k and v are the dimensions of the key and value vectors, respectively. Every input embedding attends to all other embedding using an attention head, which is computed as A.V, where *A* is an attention matrix for a particular feature. An attention matrix defines the amount of attention that should be given to the specific feature in the set of all features; Matrix *A* is calculated using the following formula:

(2) $$\begin{array}{c} A=softmax(\frac{Q*K^{T}}{\sqrt{k}}) \end{array}$$

Later, as we mentioned, the attention matrix $A\in R^{n*n}$ was multiplied by the value matrix *V* to contextually transform the embeddings into a more meaningful representation. The output of the attention head of dimension *v* was forwarded to the point-wise feed-forward layers, where it first expanded the embedding to four times (ρ) its size and then was projected back to the original embedding dimension. The final embedding can be used in multiple downstream tasks such as classification and regression.

## 4. Vocal Tab Transformer

We propose a novel transformer-based method that includes a feature-selection step to reduce the solution’s complexity and improve its overall accuracy. The proposed solution consists of the following steps.

1. Train XgBoost with the complete dataset.
2. Estimate feature importance using the trained XgBoost model.
3. Rank the features according to the importance score.
4. Select the top N features and train the proposed network

### 4.1. System Model

#### 4.1.1. Feature Selection

Tree-based models such as decision trees and the RFs are quite often used in data science for feature selection. It is quite natural to use them, as they try to keep the best-performing features closer to the root of the tree. GBDTs use the sample principle and can fit on even more complex data; specifically, GBDTs work well on unbalanced classes in comparison to RFs. As our dataset is quite complex due to the high number of features, and also because it has an unbalanced class, GBDTs seem to be the best available method for feature selection. The superiority of GBDTs over other approaches has also been corroborated empirically; experiment results are available in Table 1. Specifically, we have used Xgboost, a framework based on GBDTs. To find the relevance of the features for our application, we trained it on the complete dataset [9]. Moreover, Xgboost delivers the best result when trained with the complete set of features. During our experiments, we observed that it outperforms scikit-learn’s [45] implementation and even delivers the second-best accuracy after our proposed method. We have tried tuning the parameters of XgBoost using the Exhaustive Grid Search method available in Sklearn [45], but it turns out to be the default parameters that deliver the best result. Some important parameters are as follows:

- Booster = gbtree (Gradient Boosting Tree)
- N_estimators = 100
- Learning_rate = 0.3
- Maximum depth of a tree = 6
- Tree_method = auto

The post-training feature importance score corresponding to each feature was accessed via the inbuilt class attribute of XGBClassifier called “feature_importances_”. The “importance_type”, which was used while calculating the importance score, was “gain”, which means the average gain was calculated across all splits where the feature was used. Finally, all features were sorted according to their importance score, and the top N features were selected, which influence the outcome most. To compare the effectiveness of the feature selection strategy with other frequently available options, we selected two different strategies to compare with xgboost. These are

- The support vector classifier (SVC) feature score and
- The permutation feature score.

Similarly to the Xgboost, the SVC was trained, and the importance score was extracted for feature selection. However, for the permutation feature score, various combinations of features were selected, and their objective scores were compared using SVC. The results for the described datasets are presented in Table 1. In the subsequent sections, empirical evidence is presented to show that the model trained with the feature-selection method outperforms the model trained on the complete feature set. Another benefit of feature selection is to reduce the computational complexity of our proposed solution, which has an NN-based feature projection network for each individual feature.

#### 4.1.2. Feature Embedding

Encoding data in a language-based model are extensively studied in the field of NLP, where there are multiple well-known procedures to encode word tokens to a corresponding contextualized fixed-length vector representation. Word-embedding tools such as Word2Vec, trained on a large corpus, are also available and have been made available to use in any text-based application. Some procedures [48,49,50,51] are available to embed graphs. However, no such procedure/tool is available for the features of tabular data. Since different PD dysphonia measures come from distinct distributions, it necessitates a heterogenous embedding method.

In general, tabular data contain a mixture of categorical, ordinal, and continuous feature columns. Although categorical and ordinal features can be embedded similarly to word tokens, continuous features need a different approach due to the requirement of the linear dependency between the value of the feature and the required embedding. The success of embedding a categorical variable in tabular data is well studied and applied in TabTransformer, but there is no method defined for the continuous variable. A few other studies [52,53,54,55,56] have utilized the linear projection approach to transform continuous features to a fixed-length vector. In the PD dataset, only one variable (gender) is categorical, and the rest are continuous. Gender had an importance score of zero during the feature-selection step, which left us with only continuous features to define our solution network. Inspired by SAINT [52], we devised a linear projection-based method to embed continuous vocal features.

**Figure 3:**
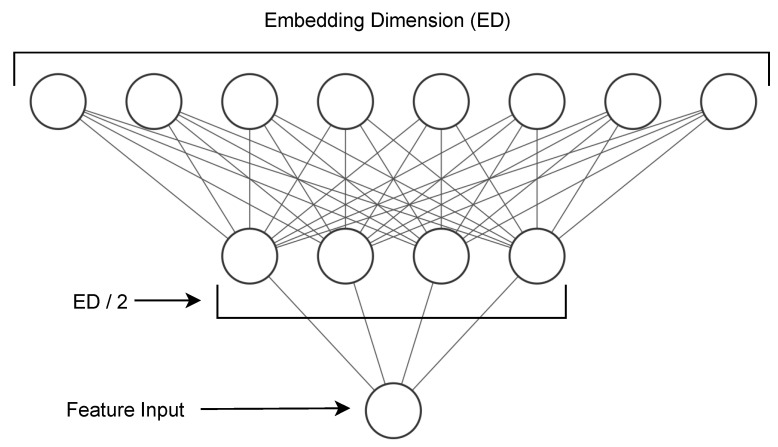
Feature embedding network to project features to fixed-length vectors. This network is made up of two fully connected layers stacked on top of each other.

Suppose $\theta ={\left(\chi_{i},y_{i}\right)}_{i=1}^{m}$ is the PD dataset with m patient records, where each consists of vocal features for a particular sample. Therefore, $\chi_{i}=\left[f_{i}^{1},f_{i}^{2},\ldots ,f_{i}^{n}\right]$ represents one patient data point with $f_{i}^{j}$ continuous features. Now, to embed each feature in a fixed-length vector *d*, we defined a separate embedding network, as shown in Figure 3, which consists of fully connected $\left[FC_{1}^{d/2}\right]$ layers to first project a single feature value to a d/2 length vector followed by a relu activation function, and we then projected it to the final required dimension *d* using another FC layer $\left[FC_{2}^{d}\right]$ followed by relu activation. At the last FC layer, we applied a dropout with a probability of 0.1 to avoid overfitting. The complete equation looks like this:

(3) $$\begin{array}{c} FE_{i}^{j}=Dropout\left(relu\left(FC_{2}^{d}\left(relu\left(FC_{1}^{d/2}f_{i}^{j}\right)\right)\right)\right) \end{array}$$

where FE stands for feature embedding.

#### 4.1.3. Transformer Block

We used a transformer encoder block to learn contextual inter-relations between the various vocal features. The vector representations derived through the feature embedding block corresponding to each feature were passed to the transformer block. A few changes were applied to the architecture. In our experiments, we observed that a higher value of ρ=32 delivered a better result. For the classification purpose, the output of the transformer encoder, let us say $E\in R^{n*k}$ is passed to the MLP head, where it is first flattened to a single row vector $R^{n*d}$. The flattened feature representation is passed to two fully connected layers followed using relu activation, which projects the vector to a smaller 2048-dimension vector and then finally to a single value output. To make output suitable for the binary classification, we have applied sigmoid activation to the final logit to squeeze the value between 0 to 1. To avoid overfitting, we have applied a dropout with a probability equal to 0.1 multiple intermediate layers.

### 4.2. Architecture and Working

Our solution contains two major building blocks, as each described in detail in the previous section:

- Feature selection
- Trainable NN model

The trainable NN model can be further classified into three major blocks:

- Feature Embedder
- Transformer Block
- MLP Head

**Figure 4:**
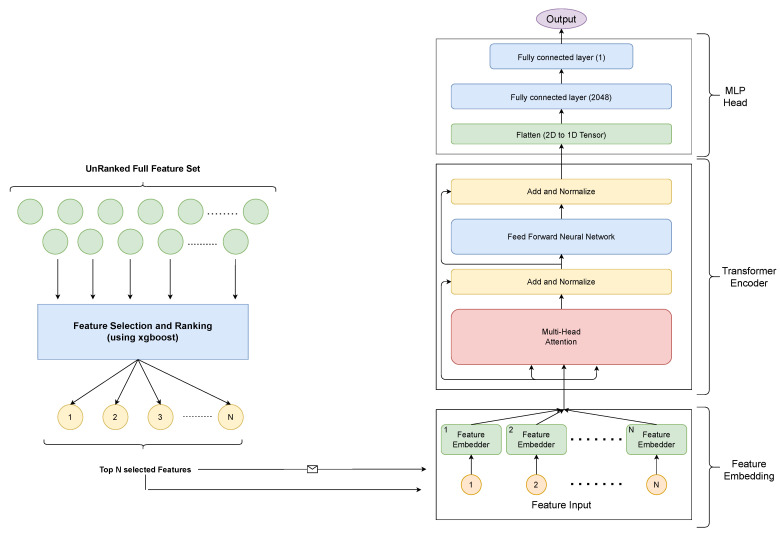
(**Left**): xgboost-based feature selection module; (**Right**): three-step solution architecture comprising the feature embedding network at the bottom followed by the transformer encoder in the center and the MLP head at the top for final classification. Modules are explained in Section 4.1.1.

The feature selection is carried out using the discussed feature selection steps, and the top N important features are selected for training and testing the proposed method. The proposed network, i.e., Vocal Tab Transformer, first consists of the feature embedder block, which transforms each feature to N-dimensional feature embedding. These features are then passed to a transformer-based encoder block, which transforms each input vector into a highly contextualized vector representation. The MLP head is used to consume this representation vector to finally predict the classification output. The architecture and the flow are illustrated in Figure 4.

## 5. Experimentation and Results

### 5.1. Experimental Setup and Parameters

#### 5.1.1. Vocal Tab Transformer

Before the models were trained, PyTorch, ADASYN, and Python were seeded with a constant random seed to make the experiments deterministic and to make comparison possible across the experiments. The proposed network was trained using the Adam optimizer [57] with binary cross-entropy loss. The configurable parameters were set to the following values after some experimentation, as explained in the subsequent section:

- Number of transformer encoders = 6
- Attention head = 1
- Feature embedding dimension = 64
- Learning rate = $8\times 10^{-4}$
- Batch size = 32
- Epoch = 20

**Figure 5:**
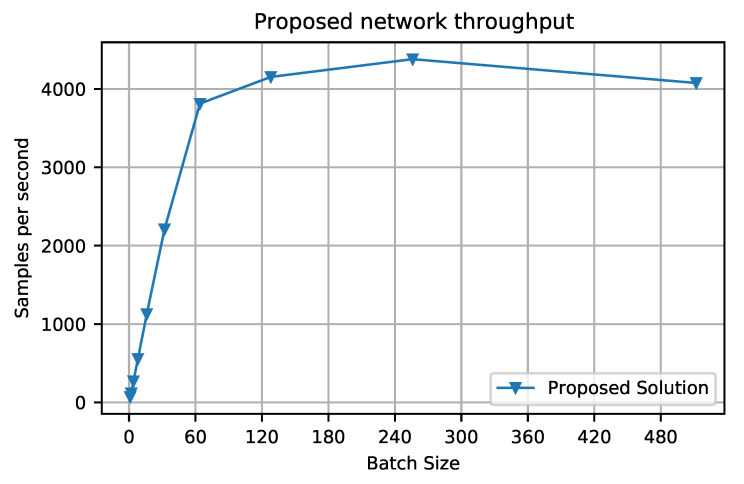
The proposed network’s throughput at different batch sizes. The model was warmed up for 100 batches, and then the throughput was calculated by using another 1000 batches.

As we have adopted the 10-fold strategy for evaluation, we have trained ten different models using one fold each and tested on the corresponding test set, and we averaged all of the AUC scores to obtain the final AUC score. This NN contains ~14 million trainable parameters, and the training and inference times are mentioned in Table 2. This model was trained on one NVIDIA GeForce RTX 2060 Max-Q GDDR6 6GB VRAM and AMD Ryzen 9 4900HS Processor. The throughput was calculated on different batch sizes to allow us to understand the network’s parallelization capacity; the results are presented in Figure 5.

#### 5.1.2. MLP

**Figure 6:**
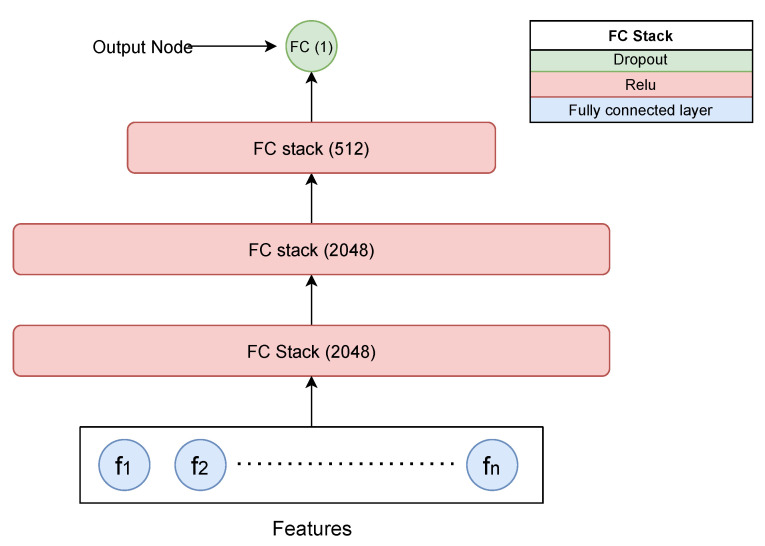
Multilayer perceptron model made up of a repeated FC stack. An FC stack is made up of a fully connected layer followed by a relu activation and finally a dropout layer. The last layer has a single node to generate the final logits.

We defined an MLP network and trained it using a similar configuration as we have used for the proposed network. Our MLP implementation contains four fully connected (FC) layers followed by a relu activation for the first three FC layers, and they are stacked sequentially, as illustrated in Figure 6. The complete feature vector $f_{i}$ is passed through the model, and the logits are then converted to classification scores using a sigmoid activation function. To avoid overfitting, we applied a dropout layer after every hidden layer with a probability of 0.1. We kept other valid configurations such as the optimizer and loss function the same as what was used to train the proposed network. This NN contains ~5.5 million trainable parameters, and the training and inference times are mentioned in Table 2. This model was trained on the same platform as used for the proposed network.

#### 5.1.3. XGBoost and Scikit-Learn’s Classifiers

The XGBoost classifier is the SOTA when it comes to working with tabular data, and it is the baseline for the proposed network (Figure 4). It is trained using the official Python implementation with the 96 input features, as we stated in the feature selection step. Similar to the feature selection step, we used the Exhaustive Grid Search method available in Sklearn to tune the hyperparameter; the only difference is that for tuning, a training set was used, while in the feature-selection step, a complete dataset was used for parameter tuning. We also tried manually calibrating the parameters and finally settled on the following, which had the best result:

- colsample_bytree = 0.3
- gamma = 0.0
- learning_rate = 0.2
- max_depth = 10
- min_child_weight = 1

There are other classifiers that researchers typically use on the dysphonia-based PD dataset and achieve SOTA performance. To compare the proposed SOTA with these frequently used methods, we used scikit-learn’s [45] implementation of the following classifiers:

- GradientBoostingClassifier [25]
- AdaBoostClassifier [45]
- RandomForestClassifier [45]
- DecisionTreeClassifier [45]
- SVM [47]
- KNeighborsClassifier [45]
- LogisticRegression [45]
- GaussianNB [45]

All of these classifiers were trained with a similar condition and input pipeline. After a few trials, we concluded that the default parameters deliver the best results for each ML algorithm. The Sklearn and Xgboost model was trained and tested on a CPU (AMD Ryzen 9 4900HS) due to its lower complexity than deep learning algorithms [58]. The average training and inference times for each algorithm are available in Table 2.

### 5.2. Results

This section comprises the results of various experiments. The list of experiments discussed here are

1. A comparison of the proposed solution with other frequently used models such as MLP, Xgboost, and RF.
2. Model performance on other datasets [23].
3. The proposed approach’s hyper-parameter effect on the AUC score.

To compare the performance of the models, we have relied on the ROC-AUC [59] score, which tells how classifiers are performing irrespective of the classification threshold. We have calculated the precision and recall scores to understand the model’s performance in-depth. To calculate the precision and recall score, we fixed the confidence threshold value to 0.5. As we have adopted a k-fold evaluation strategy with 10 folds, the ROC-AUC [59], precision, and recall scores were calculated for each set, and all scores for the respective metrics were averaged to obtain the final scores. We have discussed the data split in detail in the previous section.

All models were trained with the described feature selection, preprocessing, and oversampling method on both datasets [9,23]. The results for the two datasets are presented in Table 3 and Table 4, respectively. Each table is divided into two sections that indicate the evaluation scores with and without the feature selection step; each sections contains a distinct column for Avg ROC-AUC, precision, and recall scores from the specified model (in rows) trained on the dataset mentioned in the caption.

**Figure 7:**
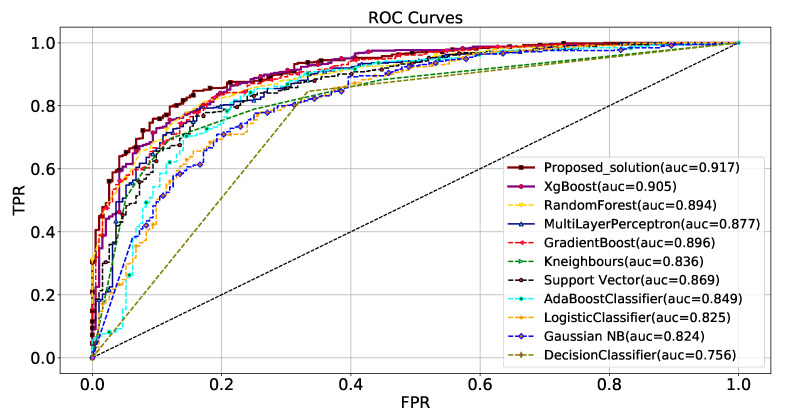
Comparative ROC curve plot of models trained on the 96 selected features from the main PD dataset. For this plot, the best models were selected from each category by comparing the k-fold average AUC score. The *Y*-axis represents True Positive Rate (TPR), whereas the *X*-axis represents False Positive Rate (FPR).

Each ROC-AUC score is reported as the mean ± standard deviation, which means the models were trained multiple times with random seeds to normalize the effect of weight initialization and other aberrant behaviors. We selected the best-performing model from each algorithm trained on the main PD dataset [9] and plotted a comparative ROC-AUC curve as shown in Figure 7.

**Figure 8:**
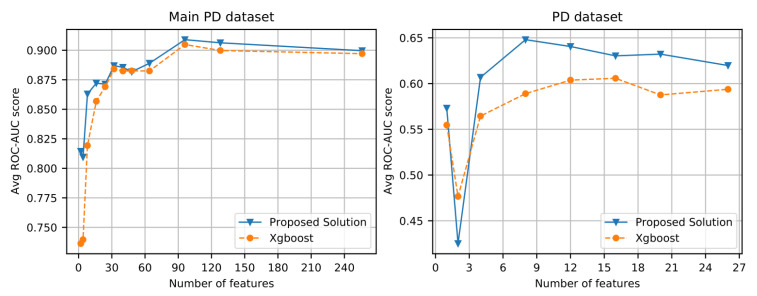
Average k-fold ROC-AUC score with respect to the number of input features in the model: (**Left**): Main PD dataset [9]; (**Right**): PD dataset [23].

To test the generalizability of the proposed approach, we considered one other dataset, i.e., PD dataset [23], whih contains multiple types of sound recordings. The AUC score on this dataset is available in Table 4. A comparison of the top two approaches, i.e., the proposed solution and the xgboost performances, on different sizes of features for the dataset used is presented in Figure 8.

To understand the effect of hyper-parameters such as attention heads, batch size, and the number of features on the performance of the proposed approach, we trained the model with different parameters. The findings are as follows.

**Figure 9:**
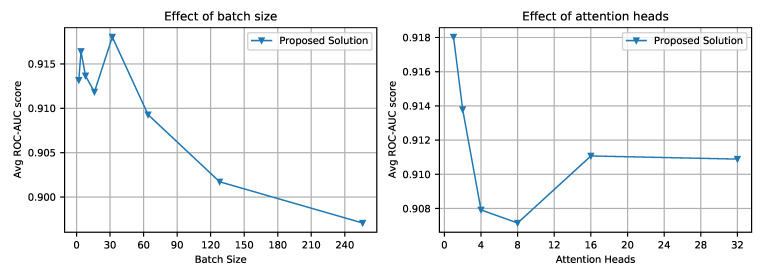
(**Left**): Performance of the proposed approach withy the different batch sizes used in the training process. (**Right**): Performance of the proposed approach with different numbers of attention heads.

1. A smaller batch size yields better results; we used 32 data points for the model training to balance the trade-off between training time and accuracy; see Figure 9 (Left).
2. The number of attention heads has no significant impact on the performance of the transformer; see Figure 9 (Right). Hence, a single attention head is selected as a default parameter for the experiments.
3. Ninety-six seems to be the right number of features to build an accurate model. There is no significant improvement when we increase the number of features is increased beyond that; see Figure 8 (Left).

**Figure 10:**
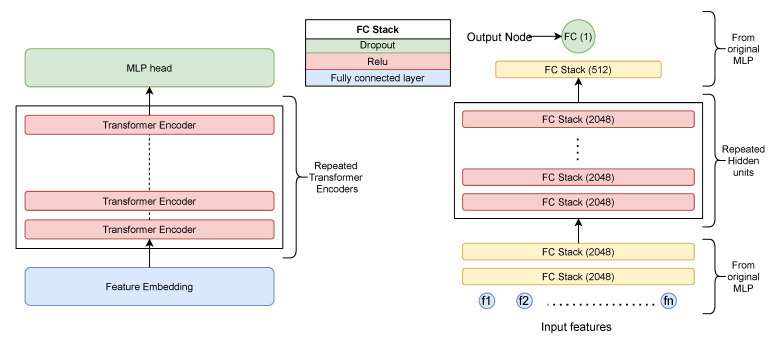
(**Left**): Modified proposed approach with multiple encoders as a hidden unit. (**Right**): Modified MLP with multiple FC stacks as a hidden unit.

We explored the option of having multiple transformer encoders on top of each other and compared the results with the increased number of hidden units in the MLP. The network configuration is illustrated in Figure 10. There is no significant improvement in the proposed network’s performance, but one empirical behavior was noticed: the proposed network’s performance remained constant, while MLP performance decreased significantly with the increased number of hidden units.

**Figure 11:**
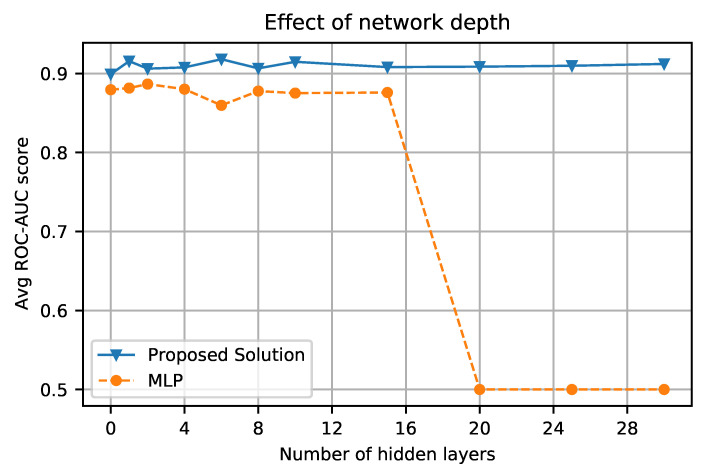
Performance comparison of the proposed approach and MLP with different numbers of hidden units present in the network. The hidden units are described in Figure 10 for the respective architectures.

The comparison graph in Figure 11 shows ROC-AUC scores compared between the proposed approach and MLP with respect to different numbers of hidden units in the respective networks.

## 6. Analysis

As we can see, the proposed approach performs best on the main PD dataset [9] as compared to the other PD dataset [23]. Despite the performance on another PD dataset [23] that contains multiple types of phonation, the proposed approach is still better than SOTA Xgboost and other ML algorithms such as Random Forest. However, the MLP on PD dataset [23] with multiple types of phonation does much better than the proposed approach and the SOTA for tabular data, e.g., xgboost, which needs further investigation to understand this aberration. One probable reason for this phenomenon is the suggested solution’s higher performance in handling complicated datasets with numerous interconnected attributes. Notably, it is clear that the effectiveness of the suggested technique improves significantly when trained on carefully chosen features rather than the entire collection of features. The presence of a self-attention layer within the suggested approach, which serves to encode individual aspects by leveraging the intermediate representations of other features, is the underlying cause of this behavior. Although an attention weight is used to determine each feature’s contribution, its efficacy appears to be less than desirable. Thus, further investigation is warranted to gain a comprehensive understanding of the distribution and impact of these attention weights.

Figure 11 shows the performance of the proposed approach in comparison to MLP’s performance with the increased depth using multiple hidden units; the performance of the proposed approach remains consistent, while MLP’s performance drops to 0.5, which means the model loses the ability to retain information with the increased depth, while the purposed solution can retain it and can learn more complex pattern if it is available. This behavior is quite similar to a computer vision architecture ResNet, where identical skip connections to the ones we have in the proposed network help the model to have more hidden units without wearing down its performance. The increased depth in ResNet allows it to perform well on a larger dataset and gives more room to learn and grasp the hidden patterns. This behavior also opens the door to the opportunity of having a more accurate model once a significantly larger PD dataset is available. In comparison, our suggested method outperforms the SOTA when the dataset is complex and contains several features, and it has a strong potential for learning hidden relationships between the retrieved features from the speech recordings. Apart from performance, it opens up the possibility of devising a system that includes multi-modality and continuous learning, which are currently unavailable with SOTA GBDTs.

## 7. Conclusions and Future Scope

Parkinson’s disease is a progressive nervous system disorder that affects movement and eventually severely affects the patient’s life by making their day-to-day activity dependent on their loved ones. Due to the worldwide surge in cases, and which are expected to rise further in the future, a system that can detect PD at an early stage is much needed for the early start of diagnosis and prevention.

We have proposed a novel approach to detect PD using dysphonia measures (vocal features) extracted from the patient’s voice recording that outperforms the current SOTA GBDT-based solution by at least 1% AUC score; the precision and recall scores are also improved. We discussed the pros and cons of the proposed solution and explained its implications in the discussion section. Moreover, the other major contributions/findings are as follows:

1. A feature selection strategy that works well with the proposed solution using XgBoost.
2. A report of a performance comparison of the frequently used ML algorithms, along with our proposed solution;
3. A novel approach to embed vocal features in fixed-length vectors using fully connected NN layers;
4. A detailed study of the different proposed network parameters and their relevance to the application; and
5. Empirical evidence of the stability of the proposed network’s performance with increased depth and a comparative study with respect to MLP, which may lead to a more accurate model once a large sample PD dataset is available.

Furthermore, an NN-based solution gives leverage over the limitations of the boosted trees and opens the door to future research for multi-modal solutions and a continual learning setup.

Our work can be extended in two different directions. (a) This method can be tested on a more extensive and diverse set of tabular datasets to find the effectiveness of this method as a general go-to approach. (b) The accuracy of PD detection can be further improved by coupling this method with KNN (as we can see, KNN has a high AUC score as well) using a constant-length vector representation generated from the transformer. Our solution can also be used in a setup similar to the Siamese network with the triplet/contrastive loss function to make the vector representation of a similar class closer by direct supervision. A study related to different augmentation strategies to avoid overfitting is also possible.

Apart from the above-mentioned works, there is also a need to study the robustness of the transformer-based network against noisy and missing data and the interpretability of the contextual embeddings of the vocal features. 

## Figures and Tables

**Table 1:** The proposed solution’s AUC-ROC score with respect to the different feature selection strategies on the mentioned datasets.

Method	Main PD Dataset [9]	PD Dataset [23]
XgBoost [46]	0.91432 ± 0.0037	0.64649 ± 0.0082
Support Vector Classifier [47]	0.88379 ± 0.0156	0.60249 ± 0.0002
Permutation [45]	0.82015 ± 0.1062	0.58297 ± 0.0090

**Table 2:** Training and inference time for each model in milliseconds (ms). Training time was calculated for each training set in k-fold splits, and then the average train time was calculated. Inference time was calculated on a batch of 78 samples.

Model	Training Samples	Avg Training Time	Test Samples	Avg Inference Time	Avg Inference Time per Sample
Proposed network (Figure 4)	678	38,860.778	78	1029.443	13.197
MLP (Figure 6)	678	2586.155	78	179.195	2.297
Xgboost [46]	678	537.893	78	3.362	0.043
GradientBoosting [25]	678	15,637.271	78	0.417	0.005
AdaBoost [45]	678	3530.252	78	6.293	0.080
RandomForest [45]	678	1385.690	78	8.577	0.109
DecisionTree [45]	678	848.363	78	0.309	0.003
SVM [47]	678	1499.896	78	23.164	0.296
KNeighbors [45]	678	1.022	78	8.181	0.104
LogisticRegression [45]	678	171.001	78	0.208	0.002
GaussianNB [45]	678	4.369	78	0.682	0.008

**Table 3:** Average ROC-AUC, precision, and recall scores for each model on the main PD dataset [9]. The results are shown with and without the feature selection phase. Scores are presented as the mean and standard deviation.

Model	With Feature Selection (96 Features)			Without Feature Selection (753 Features)		
	Avg ROC-AUC	Avg Precision	Avg Recall	Avg ROC-AUC	Avg Precision	Avg Recall
Proposed network	0.9143 ± 0.0037	0.8819 ± 0.0043	0.90378 ± 0.0103	0.8574 ± 0.0039	0.8234 ± 0.0069	0.8421 ± 0.004
Xgboost	0.9028 ± 0.0042	0.8634 ± 0.0021	0.8693 ± 0.0074	0.875 ± 0.0041	0.8421 ± 0.0078	0.8411 ± 0.0039
MLP	0.8728 ± 0.0039	0.8328 ± 0.0095	0.8712 ± 0.0068	0.82565 ± 0.0130	0.8032 ± 0.0094	0.7908 ± 0.0124
GradientBoosting	0.9009 ± 0.0008	0.8634 ± 0.0021	0.8584 ± 0.0083	0.87083 ± 0.0024	0.8414 ± 0.0028	0.841 ± 0.0067
AdaBoost	0.8546 ± 0	0.8584 ± 0	0.8514 ± 0	0.85756 ± 0	0.8523 ± 0	0.8544 ± 0
RandomForest	0.8939 ± 0.0041	0.7981 ± 0.006	0.8643 ± 0.0092	0.85917 ± 0.0053	0.8251 ± 0.0063	0.803 ± 0.0067
DecisionTree	0.7456 ± 0.0072	0.7213 ± 0.0082	0.7749 ± 0.001	0.69195 ± 0.0082	0.6642 ± 0.0149	0.6597 ± 0.0212
SVM	0.8737 ± 0	0.8031 ± 0	0.8723 ± 0	0.80743 ± 0	0.7731 ± 0	0.7674 ± 0
KNeighbors	0.84047± 0	0.8031 ± 0	0.8599 ± 0	0.7796 ± 0	0.7438 ± 0	0.7264 ± 0
LogisticRegression	0.83081 ± 0	0.8321 ± 0	0.794 ± 0	0.78466 ± 0	0.777 ± 0	0.776 ± 0
GaussianNB	0.83593 ± 0	0.7816 ± 0	0.7943 ± 0	0.76863 ± 0	0.7422 ± 0	0.7374 ± 0

**Table 4:** Average ROC-AUC, precision, and recall scores for each model on the another PD dataset [23]. The results are shown with and without the feature selection phase. Scores are presented as the mean standard deviation.

Model	With Feature Selection (8 Features)			Without Feature Selection (26 Features)		
	Avg ROC-AUC	Avg Precision	Avg Recall	Avg ROC-AUC	Avg Precision	Avg Recall
Proposed network	0.6464 ± 0.0024	0.623 ± 0.0035	0.6304 ± 0.0027	0.6293 ± 0.0081	0.6034 ± 0.0023	0.6013 ± 0.0018
Xgboost	0.5761 ± 0.0104	0.5532 ± 0.0076	0.5527 ± 0.0038	0.568 ± 0.0021	0.5521 ± 0.0076	0.562 ± 0.054
MLP	0.6920 ± 0.0062	0.6439 ± 0.0103	0.6134 ± 0.0089	0.6605 ± 0.0040	0.6532 ± 0.0061	0.6243 ± 0.0095
GradientBoosting	0.5992 ± 0.0003	0.542 ± 0.0001	0.5565± 0.0003	0.5748 ± 0.0014	0.5613 ± 0.0009	0.5443 ± 0.001
AdaBoost	0.5932 ± 0	0.5824 ± 0	0.5703 ± 0	0.5403 ± 0	0.5272 ± 0	0.5326 ± 0
RandomForest	0.6086 ± 0.0058	0.5554 ± 0.0017	0.5472 ± 0.0016	0.5737 ± 0.0050	0.5523 ± 0.0094	0.5145 ± 0.0019
DecisionTree	0.5147 ± 0.0048	0.4824 ± 0.0136	0.4621 ± 0.0104	0.5375 ± 0.0040	0.5124 ± 0.0103	0.4924 ± 0.0048
SVM	0.6176 ± 0	0.5824 ± 0	0.5578 ± 0	0.5937 ± 0	0.5434 ± 0	0.5251 ± 0
KNeighbors	0.5953 ± 0	0.5627 ± 0	0.5936 ± 0	0.5836 ± 0	0.5421 ± 0	0.5738 ± 0
LogisticRegression	0.6307 ± 0	0.6131 ± 0	0.6014 ± 0	0.606 ± 0	0.5839 ± 0	0.5982 ± 0
GaussianNB	0.5832 ± 0	0.6021 ± 0	0.5341 ± 0	0.5705 ± 0	0.5894 ± 0	0.5474 ± 0

## Data Availability

Not applicable.
